# Hopkins syndrome following the first episode of bronchial asthma associated with enterovirus D68: a case report

**DOI:** 10.1186/s12883-018-1075-7

**Published:** 2018-05-23

**Authors:** Fumie Hayashi, Shintaro Hayashi, Dai Matsuse, Ryo Yamasaki, Keiji Yonekura, Jun-ichi Kira

**Affiliations:** 1 Department of Neurology, Neurological Institute, Graduate School of Medical Sciences, Kyushu University, 3-1-1 Maidashi, Higashi-ku, Fukuoka, 812-8582 Japan; 2 Department of Pediatrics, Kure Medical center, Hiroshima, Japan

**Keywords:** Hopkins syndrome, Bronchial hyper-reactivity, Intravenous immunoglobulin, Enterovirus D68, First asthma attack

## Abstract

**Background:**

Hopkins syndrome (HS) is a rare disorder presenting with acute flaccid paralysis of the limbs following an asthma attack. Neurologists encounter a diagnostic challenge if patients without a history of bronchial asthma develop neurologic features mimicking HS following acute respiratory distress. We report a case of HS occurring after a first episode of bronchial asthma associated with enterovirus D68 infection.

**Case presentation:**

A 5-year-old girl developed acute respiratory distress. On the fourth hospital day, both her legs became paralyzed except for slight muscle contraction in the right lower limb. Tendon reflexes in the lower limbs were diminished and there was a positive Babinski sign on the right. Sensation was normal in all modalities, and there was no uro-rectal disturbance. Spinal magnetic resonance imaging identified T2-hyperintense lesions with spinal cord edema, mainly involving the bilateral T11 to L1 anterior horns, with left side dominance extending to the left posterior horn. The neurological and neuro-radiological findings of our case were suggestive of HS; however, she had no history of bronchial asthma. An acetylcholine inhalation challenge eventually proved the presence of reversible airway hyper-responsiveness, allowing us to diagnose HS. We identified enterovirus D68 in the patient’s intratracheal aspirates using a sensitive polymerase chain reaction assay. Intravenous immunoglobulin administrations at 2 g/kg^2^ for 5 consecutive days were repeated every month up to four times. After these treatments, the muscle strength of her right lower limb slightly improved while her left lower leg remained completely paralyzed.

**Conclusion:**

This case emphasizes the importance of provocation tests to reveal the presence of airway hyper-responsiveness when a child shows neurological signs mimicking HS following acute respiratory distress. Furthermore, the present case suggests a possible link between HS and acute flaccid paralysis following lower respiratory tract infection by enterovirus D68.

## Background

Hopkins syndrome (HS) is a poliomyelitis-like illness that occurs acutely after an asthmatic attack. It was first reported in 1974 [1], and so far only about 40 cases have been reported. HS mostly affects children, typically occurs within several days to a few weeks following an acute asthmatic attack, and presents as flaccid paralysis of one or more extremities. Due to its rarity, the pathogenesis and effective therapy of HS remains to be elucidated. We report a case of HS occurring after a first episode of bronchial asthma (BA), associated with enterovirus D68 infection, in which the presence of airway hyper-responsiveness was confirmed by provocation tests.

## Case presentation

A healthy 5-year-old girl developed acute respiratory distress and became unconscious. On arrival at the Emergency Room, venous blood gas analysis showed type II respiratory failure and respiratory acidosis (pH 7.011, partial pressure of O_2_ 93.7 mmHg, partial pressure of CO_2_ 112.0 mmHg, serum bicarbonate concentration 27.0 mmol/l, base excess − 7.3 on room air). She was mechanically ventilated under midazolam sedation. Initially, anaphylactic shock of unknown origin was suspected. Epinephrine (0.03 mg/kg) was administered subcutaneously, leading to partial improvement in tidal volume. Serum immunoglobulin E (IgE) concentration was elevated (166 IU/ml, normal < 70 IU/ml for her age), and specific IgE to house dust mites was strongly positive (*Dermatophagoides pteronyssinus* 91.8 UA/ml; *Dermatophagoides farinae* > 100 UA/ml).

**Fig. 1:**
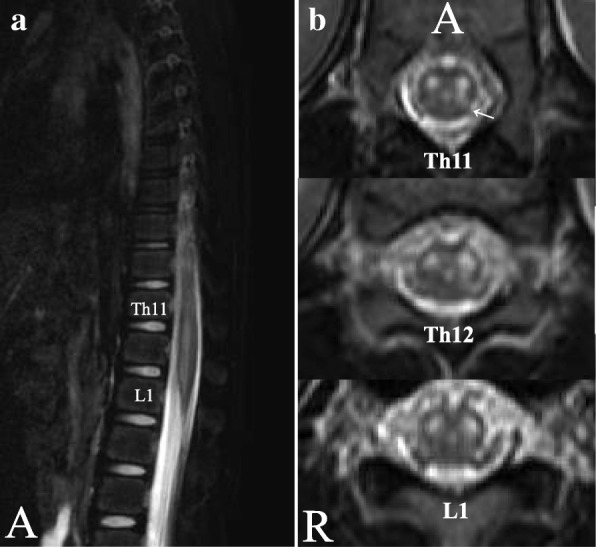
**a** Sagittal T2-weighted magnetic resonance image showing a hyperintense lesion and spinal cord edema continuously from the T11 to L1 spinal levels; **b** axial T2-weighted images at T11, T12 and L1 show hyperintense lesions in both anterior horns, worse on the left, with left posterior horn involvement (arrow). Key: R, right; A, anterior

Although the patient had no history of BA, a first asthma attack was suspected, and methylprednisolone (2 mg/kg/day) and ampicillin/sulbactam (100 mg/kg/day) were given. Serologic tests for infection on the 2nd day after the onset of respiratory distress, including echovirus, enterovirus, coxsackievirus, poliovirus, herpes simplex virus, and *Mycoplasma* were negative. On the 4^th^ day after symptom onset, she was weaned off mechanical ventilation and extubated. At this time, when her level of consciousness improved, her legs showed no paralysis. However, a few hours later when she woke from sleep, both her legs were paralyzed except for weak motor activity in the right anterior tibialis muscle. Patellar and Achilles tendon reflexes were diminished; there were no pathologic reflexes. Cerebrospinal fluid (CSF) analysis on the 21st day after symptom onset showed 7 cells/μl (100% mononuclear cells), elevated protein concentration (120 mg/dl, normal 15–40 mg/dl), and normal myelin basic protein concentration. Virus isolation culture did not detect any viruses in the CSF. Spinal magnetic resonance imaging (MRI) on the 21st day after symptom onset identified T2-hyperintense lesions with spinal cord edema, mainly involving the anterior horns bilaterally from T11 to L1, with left side dominance extending to the left posterior horn (Fig. 1). On the 23rd day after the onset, HS was suspected and intravenous immunoglobulin (IVIg) (400 mg/kg/day for 5 days) followed by intravenous methylprednisolone (30 mg/kg/day for 3 days) were administered, but there was no improvement over the next 3 weeks.

On the 47th day after symptom onset, she was transferred to our institution. She was alert and orientated, and her cranial nerves were intact. She had diffuse muscle atrophy in the left lower limb, with diminished tone but no fasciculation. Marked muscle weakness was evident in iliopsoas (Medical Research Council [MRC] scale grade right/left 0/0), the thigh adductors (0/0) and abductors (0/0), gluteus maximus (0/0), quadriceps femoris (0/0), biceps femoris (0/0), anterior tibialis (3/0), gastrocnemius (3/0), and dorsi- (3/0) and plantar-flexors of the toes (3/0). Tendon reflexes in the lower limbs were diminished. The Babinski sign was positive on the right. Sensation was normal in all modalities, and there was no uro-rectal disturbance. Complete blood count, including eosinophil count, blood biochemistry, coagulation status, and the concentrations of D-dimer, proteins S and C and lupus anticoagulant were normal. Serum myeloperoxidase and proteinase-3 anti-neutrophil cytoplasmic antibodies were negative, and autoantibodies against aquaporin-4 and phospholipid-bound β2–glycoprotein I were absent. Enterovirus D68 was detected in endotracheal aspirates (obtained on the 2nd day after symptom onset), but not in the CSF (obtained on the 21st day after onset), using a consensus-degenerate hybrid oligonucleotide primer polymerase chain reaction (PCR) method with direct sequencing,. Nerve conduction studies identified decreased compound muscle action potential amplitudes in the left tibial nerve (0.04 mV, normal > 2.9 mV). There was no evidence of arteriovenous malformation or arteriovenous fistula on spinal MR angiography. With parental permission, an acetylcholine (ACh) bronchial stimulation test was undertaken by a pulmonologist, which showed that inhalation of 5000 μg/ml ACh provoked wheezing and a rapid reduction in forced expiratory volume from 0.86 l/min to 0.50 l/min and a flow-volume loop typical of peripheral airway obstruction, both of which were reversed by 1.0 mg inhaled salbutamol. These findings were diagnostic of BA, and consequently HS.

Intravenous immunoglobulin 0.4 g/kg was administered for five consecutive days a month for 4 months, based on previous case reports showing its efficacy in HS [2, 3]. After treatment, power in the right anterior tibialis, dorsi- and plantar-flexors of the toes and gastrocnemius muscles improved to MRC grade 4, but power remained MRC grade 0 throughout the left lower limb. Spinal MRI undertaken on the 103rd day after symptom onset showed atrophy of the left anterior horn. She was transferred to another hospital for rehabilitation.

## Discussion and conclusion

In this patient, airway hypersensitivity provoked by ACh inhalation and reversed by a β-adrenoreceptor agonist together with atopy to *Dermatophagoides* suggests that her episode of acute respiratory distress was the first manifestation of BA [4]. Consequently, we judge her diagnosis to be HS following a first episode of BA associated with enterovirus D68 infection. Besides flaccid paraparesis due to lumbar anterior horn cell involvement, the patient had right pyramidal tract signs and left posterior horn involvement on MRI. Involvement of the pyramidal tracts and posterior horns have also been reported in HS [5, 6].

Although the pathogenesis of HS is not understood, infectious and immune mechanisms are thought to be responsible [2, 5, 7, 8]. We identified enterovirus D68 in our patient’s intratracheal aspirates using a sensitive assay, even though serum anti-enterovirus antibodies were negative. Intriguingly, a series of cases of acute flaccid paralysis was reported after an outbreak of lower respiratory tract infection by enterovirus D68 in the USA in 2014 [9, 10]. In a nationwide survey undertaken in Japan in 2015, three out of 205 individuals diagnosed with enterovirus D68 infection exhibited flaccid paralysis (one of whom was our patient) (https://www.niid.go.jp/niid/en/iasr-vol37-e/6283-inx432-e.html). Both our and the former cases [9, 10] are similar in that they showed severe paralysis and longitudinally extending spinal cord lesions of the central grey matter with predominant anterior horn involvement. However, the current case also had posterior horn involvement and no brainstem lesions, which distinguishes it from the cases with enterovirus D68 infection-related flaccid paralysis [9, 10]. Unfortunately, provocation tests to reveal the presence of airway hyper-responsiveness were not performed in any of the former cases with enterovirus D68 infection-related flaccid paralysis. Negative findings for enterovirus D68 in our patient’s CSF do not support direct CNS invasion by the virus, but enterovirus D68 infection in the context of BA causing acute respiratory distress appears to have been the trigger for HS. The effectiveness of repeated IVIg in this case supports an immune-mediated mechanism for the pathogenesis of her flaccid myelitis, although the natural progression of the disease course is also a plausible explanation for her limited amelioration.

Alternatively, it is possible that the patient developed acute flaccid paralysis in the context of enterovirus D68 respiratory infection, which became particularly severe because she had asthma. Even in this case, we consider that IVIg administration is reasonable for treating acute flaccid paralysis caused by EVD68. IVIg contains neutralizing antibodies to EVD68 [11, 12] and has been shown to be effective in experimental animal infection by EVD68 [13] and in a patient with acute flaccid myelitis associated with EVD 68 infection [14]. Thus, the diagnosis of asthma is not pre-requisite for deciding the indication of immunotherapy in the present, and similar, cases.

Given that a number of viruses can cause respiratory illness, to perform PCR targeting for enterovirus D68 in all patients with solely respiratory symptoms might not be acceptable to all clinicians. However, to detect enterovirus D68 prior to full development of paralysis is of great significance to avoid devastating sequelae, as seen in our patient and previously reported children with enterovirus D68 infection-related flaccid paralysis [9, 10]. Therefore, evaluating the presence of enterovirus D68 and strain differences [14] in both the CSF and intratracheal aspirates from patients with respiratory illness followed by paralysis is warranted in future to clarify any pathogenic roles. Additionally, development of more sensitive assays to detect enterovirus D68 in CSF may further illuminate the involvement of enterovirus D68 in HS.

## Abbreviations

ACh: Acetylcholine
BA: Bronchial asthma
CSF: Cerebrospinal fluid
HS: Hopkins syndrome
IgE: Immunoglobulin E
IVIg: Intravenous immunoglobulin
MRC: Medical Research Council
MRI: Magnetic resonance imaging
PCR: Primer polymerase chain reaction
